# The brain age gap as a predictor of alcohol initiation in adolescence

**DOI:** 10.1016/j.dcn.2026.101793

**Published:** 2026-08-02

**Authors:** H. Byrne, R. Visontay, E.K. Devine, N.E. Wade, J. Jacobus, A.J. Moore, L.M. Squeglia, L. Mewton

**Affiliations:** a The Matilda Centre for Research in Mental Health and Substance Use, The University of Sydney, Sydney, NSW, Australia; b Department of Psychiatry, University of California San Diego, La Jolla, CA, United States; c Department of Psychiatry and Behavioral Sciences, Medical University of South Carolina, Charleston, SC, United States

## Abstract

**Background:**

Growing evidence suggests regional and network-level brain imaging features in late childhood are predictive of alcohol use in adolescence. However, the directionality of these effects (i.e. whether they reflect accelerated or delayed neuromaturation) are mixed. We applied a Brain Age Gap Estimation (BrainAGE) model to examine whether overall deviations from typical brain aging trajectories are predictive of (1) alcohol initiation and (2) use behaviour (experimentation versus binge drinking) in adolescence.

**Methods:**

Data from the Adolescent Brain Cognitive Development study release 6.0 were used. Baseline (ages 9–11) structural imaging features (cortical volume, area, and subcortical volume) were used to estimate BrainAGE. Alcohol use was determined using self-report data from the Substance Use Interview and Timeline Follow-Back across follow-ups (waves 1–6; ages 10–17). Logistic generalized mixed effects models examined whether BrainAGE predicted group status between (1) non-initiators (n = 3639) and initiators (n = 1176), and; (2) experimentation (at least one full drink, no binge episodes; n = 461) and binge drinking (at least one episode; n = 438).

**Results:**

When adjusting for age, sex, and pubertal status, a one-standard-deviation decrease in BrainAGE (equivalent to 1.64 years) at baseline was associated with a 9.5% increase in odds of alcohol initiation in adolescence. However, this effect did not survive adjustment for sociodemographic and prior alcohol exposure covariates. Further, BrainAGE did not discriminate between experimentation and binge drinking.

**Conclusions:**

Findings suggest BrainAGE in late childhood may reflect potential risk for alcohol initiation, but not behaviours, in adolescence. However, this association likely reflects complex interactions between brain structure and broader contextual factors, rather than a direct effect of brain structure alone.

## 1 Introduction

Alcohol use in adolescence poses a significant public health concern. Recent data from the World Health Organization shows 57% of adolescents reporting alcohol use before age 16, and 20% reporting having experienced significant drunkenness (more than one binge drinking episode) by this age (Charrier et al., 2024). Earlier alcohol initiation is associated with increased risk of adverse outcomes across the lifespan, including subsequent heavy drinking and substance use disorders (Gardner et al., 2024, Lee et al., 2024), poorer academic performance (Hjarnaa et al., 2023), greater risk-taking behaviours (Clare et al., 2026), and increased mental health and social difficulties (Tackaberry-Giddens et al., 2023). Because adolescence is a sensitive period for neurodevelopment, alcohol exposure during this time can also disrupt maturation of neural systems involved in executive function, reward processing, and emotion regulation (Spear, 2018), making adolescent alcohol use particularly consequential. As a result, clarifying the risk factors and mechanisms underlying alcohol initiation is critical for informing prevention efforts and early intervention during this age period.

While the impacts of alcohol use on the brain have been well documented, evidence increasingly suggests distinct neurobiological characteristics may predate alcohol use. Specifically, research suggests aberrant development of subcortical and frontal systems prospectively predicts early initiation and escalation of alcohol use in adolescence (Lees et al., 2021), aligning with hypotheses suggesting neurobiological correlates of addiction may reflect both causes and consequences (Bogdan et al., 2023, Miller et al., 2024). This is supported by recent studies conducted in large, population imaging datasets such as the Adolescent Brain Cognitive Development (ABCD) study that have identified structural morphometric properties at both the regional (Miller et al., 2024, Green et al., 2024) and network (Byrne et al., 2026) level in late childhood (ages 9–11) are predictive of alcohol use behaviours in early adolescence (up to age 15). However, the directionality of these effects – specifically, whether these observations reflect ‘accelerated’ or ‘delayed’ neuromaturation – have yet to be determined. Further, it remains unclear the extent to which alcohol use behaviours, particularly experimentation compared to binge drinking, are differentially related to structural brain properties.

Currently, few analytical methods are available to robustly assess maturation in the developing adolescent brain. One approach is brain age gap estimation (often abbreviated as ‘BrainAGE’ or ‘BAG’), a neuroimaging-based metric that computes the difference between an individual's predicted brain age – derived from machine learning models applied to brain-imaging measures – and their chronological age. Notably, while these models do not provide an accurate prediction of maturation, BrainAGE can offer a general indicative measure of deviations from typical brain aging trajectories (Whitmore and Beck, 2025). In this context, a positive gap is interpreted as the brain appearing “older” than expected in comparison to the normative or control sample, while a negative gap points to the brain appearing “younger”. BrainAGE has been frequently applied within the adolescent and adult alcohol literature to investigate the impacts of alcohol use on deviations from normative developmental trajectories, which have typically identified a positive (higher) BrainAGE following chronic and/or heavy alcohol use (Scheffler et al., 2024). However, the predictive utility of BrainAGE in the initiation and type of alcohol use behaviour (experimentation vs. binge drinking) remains unknown.

Using data from the ABCD study, the current study aims to investigate whether BrainAGE in late childhood (baseline; ages 9–11) can predict alcohol initiation in mid adolescence (wave 6; ages 15–17). Next, this work aims to examine whether baseline BrainAGE can differentiate between adolescents who have experimented with alcohol (defined here as consumption of a full drink but no binge drinking) compared to those who report at least one binge drinking episode.

## 2 Methods and materials

### 2.1 Ethics approval and consent to participate

Data from the ABCD Study release 6.0 were used, which includes behavioral and biological data from 11,868 participants aged 9–11 years at baseline. Participants were recruited from 21 sites across the United States between September 2016 and November 2018, with follow-up visits conducted at yearly intervals. Parents/caregivers provided signed informed consent and all participants gave assent. The ABCD protocol was approved by the centralized institutional review board (IRB) at the University of California, San Diego and by the IRBs at the 21 sites. For secondary analysis of de-identified ABCD data in the current study, no additional ethical approval was required. All methods were performed in accordance with the relevant guidelines and regulations.

### 2.2 Participants

Release 6.0 contains data from baseline through to wave 6 follow-up, with varying numbers of missed visits across follow-ups and data collection still ongoing at this stage. As data at the final available follow-up (wave 6; ages 15–17) aligns with the critical window for adolescent alcohol initiation (Swendsen et al., 2012), we included all available data through to wave 6 (n = 5056). Participants who had not initiated alcohol use were retained only if they had data at wave 6, whereas initiators were included if initiation occurred at any point prior to or at wave 6. To evaluate whether non-initiators had sufficient time to initiate, we compared their age distribution at wave 6 with the age-at-first-use distribution among initiators (Figure S1). The overlap in these distributions suggests that the non-initiators included in these analyses were comparable in age to the alcohol initiation group, supporting the use of this approach for classification.

**Fig. 1:**
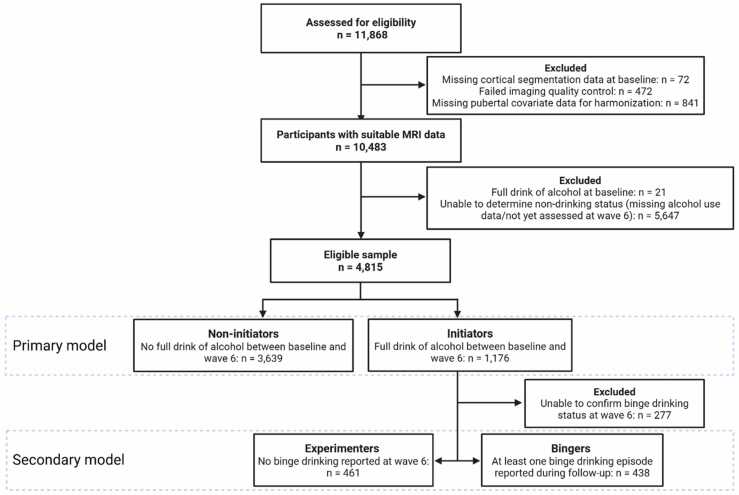
Flowchart for participant inclusion/exclusion criteria.].

For BrainAGE calculation, participants were excluded if they were missing structural MRI data at baseline (n = 72), did not meet neuroimaging quality-control criteria (n = 472), or lacked complete covariate information required for harmonization of imaging data across scanners (n = 841; see *Image Processing*). For group classification, participants were excluded if they reported consuming a full alcoholic drink at baseline (n = 21); if non-drinking status within the non-initiator group could not be confirmed at wave 6 (i.e. missing data/not yet tested at wave 6; n = 5647; see *Alcohol use classification*); if binge drinking status within the experimenter subgroup could not be determined at wave 6 (n = 277; see *Alcohol use classification*). Of the 11,868 participants recruited at baseline, 4815 met inclusion criteria (Fig. 1; n = 1176 initiators).

## 3 Measures

### 3.1 Image processing

Preprocessed imaging data acquired at baseline (ages 9–11) were used (Hagler et al., 2019). Cortical parcellation and subcortical segmentation of T1-weighted structural imaging data were performed using FreeSurfer (v7.1.1; https://surfer.nmr.mgh.harvard.edu/). Postprocessed cortical volume and area measurements mapped to the Desikan-Killiany atlas included 34 cortical parcellations per hemisphere, for a total of 68 regions of interest (ROIs) (Desikan et al., 2006), respectively, and 53 bilateral global and subcortical volumetric measures derived from the FreeSurfer output.

To remove unwanted technical variability while preserving biological signals, neuroimaging measures were harmonized across scanners using ComBat (v1.0.13; Fortin et al., 2018). Scanner characteristics (device serial number) was modelled as batch covariates, while chronological age, sex, and pubertal status (derived from parent-report Pubertal Development Scale scores; see *Pubertal status*) were preserved as biological covariates of interest.

### 3.2 BrainAGE model

To estimate BrainAGE, we used the pretrained developmental model by Drobinin et al. (2022), selected for its strong external validation andfavourable performance in a systematic evaluation of BrainAGE algorithms in youth cohorts (Michael et al., 2026). While the Drobinin model was trained across a broader age range (9–19 years) than the current sample (9–11 years), it has been evaluated in baseline ABCD participants by Whitmore et al. (2023) (n = 11,402, ages 9–11 years), demonstrating acceptable predictive performance (raw MAE = 2.32 years; bias-adjusted MAE = 1.45 years). Whitmore et al. (2023) further determined that BrainAGE estimates derived from the model were associated with pubertal development and showed moderate longitudinal stability, providing support for their use as an indicator of developmental variation. Although Whitmore et al. (2023) also retrained the model using ABCD data and achieved lower raw MAE, the original Drobinin et al. model was applied directly to baseline ABCD structural MRI data in the current study without retraining to preserve the independence of the BrainAGE estimates from model training in ABCD, reduce the risk of dataset-specific optimisation, and facilitate comparison with applications of the model in external cohorts.

The Drobinin model was trained on 1299 participants aged 9–19 years, drawn from six independent non-ABCD samples. Age and sex distributions for each sample are reported in Drobinin et al. (2022). Participants were included in model training if they fell within the 9–19-year age range, passed automated MRI quality-control procedures, had no psychiatric diagnoses, and had an IQ greater than 75. Notably, the normative training sample was not explicitly screened for alcohol or substance use exposure, which may introduce some heterogeneity into the developmental trajectories captured by the model, and is acknowledged as a limitation of the current approach. Model training was conducted within the *tidymodels* (version 0.1.1) framework, using the eXtreme Gradient Boosting (XGBoost) machine learning algorithm (version 1.0.0.2). Scan age in the Drobinin model was predicted from 189 structural imaging features, including cortical gray matter volume and surface area measurements, as well as bilateral global and subcortical volume measurements. Cortical features were obtained using FreeSurfer cortical parcellations mapped to the Desikan-Killiany atlas, while bilateral global and subcortical volume measures were obtained using default FreeSurfer segmentation output (aseg). The Drobinin model reported a mean absolute error (MAE; the average absolute difference between predicted and chronological age, where prediction errors of approximately 1–2 years are commonly observed in developmental samples (Whitmore and Beck, 2025) of 1.53 years in typically developing adolescents. This performance generalized to both the held-out validation sample (MAE = 1.55 years, bias-adjusted MAE = 1.98 years) and the independent at-risk datasets (MAE = 1.49 years, bias-adjusted MAE = 1.86 years).

While the Drobinin et al. (2022) model was originally trained on 189 structural features, only 175 of these features were available in the ABCD dataset. Therefore, for the current study, the model was used to predict BrainAGE based on the 175 available features in ABCD, replicating the methods by Whitmore et al. (2023). Consistent with Whitmore et al. (2023), the 14 unavailable features were coded as NA, which is handled natively by the XGBoost algorithm underlying the Drobinin et al. (2022) model. Missing features comprised global brain volume summary metrics, brain mask and intracranial volume ratio measures, and volumes of small peripheral structures (bilateral vessel, choroid plexus, and optic chiasm volumes), rather than direct regional cortical or subcortical morphometric measures, and were therefore considered unlikely to substantially impact BrainAGE estimation. A list of included features can be found at https://github.com/hbyrne07/brainage-in-adolescent-alcohol-initiation. BrainAGE was estimated on the full harmonized sample (n = 10,483) prior to group classification.

### 3.3 Bias correction

Consistent with prior work (Le et al., 2018, Smith et al., 2019, Whitmore et al., 2023), bias correction was applied to account for systematic over- or underestimation related to age. Specifically, slope and intercept parameters derived from the validation procedure by Drobinin et al. (2022) were applied directly to the ABCD sample to preserve consistency with the pretrained model implementation, as implemented in Whitmore et al. (2023). Age was also included as a covariate in all subsequent statistical analyses to further control for residual age-related bias, as recommended in other work (Whitmore and Beck, 2025, Peng et al., 2021), and age outliers (participants aged ≤8.9 or ≥11.1; n = 3) were removed.

## 4 Alcohol use classification

### 4.1 Alcohol use

Participants were classified into alcohol use groups using a hierarchical approach based on longitudinal self-reported data from the Substance Use Interview (SUI) and Timeline Follow-Back (TLFB) modules. Participants lacking complete SUI or TLFB data as per our exclusion criteria were excluded.

### 4.2 Primary model classification (non-initiators vs. initiators)

‘Non-initiators’ (n = 3639) were defined as participants who did not report consumption of a full alcoholic drink in any session prior to and including wave 6. Participants with insufficient data at wave 6 to confirm non-initiation status at this timepoint were excluded. ‘Initiators’ (n = 1176) were defined as participants who reported consuming at least one full alcoholic drink in any follow-up session before wave 6. This was assessed using binary ‘Yes’/‘No’ participant responses to the question: “Have you used since we last saw you: A full drink of beer, wine or liquor (rum, vodka, gin, whiskey),” as self-reported during the SUI.

### 4.3 Secondary model classification (experimenters vs. binge drinkers)

Among participants classified as ‘Initiators’, ‘Bingers’ (n = 438) were defined as participants who reported at least one binge drinking episode at any timepoint during follow-up. Binge episodes were defined as ≥ 4 standard drinks for females or ≥ 5 for males in a single drinking session, as self-reported in the TLFB. ‘Experimenters’ (n = 461) were defined as participants who reported a full drink of alcohol by wave 6 with no reported binge drinking episodes at any session prior to wave 6. To ensure Experimenters were appropriately characterized, Experimenters were required to have data available at wave 6 confirming non-binge drinking status at this timepoint (n = 277 excluded).

### 4.4 Covariates

Previous research has identified robust associations between age, sex, and pubertal status, respectively, in BrainAGE estimates (Whitmore et al., 2023, Holm et al., 2023a). Therefore, these variables were included as primary covariates in the study. Chronological age and sex at baseline were determined from parent-reported date of child’s birth and sex assigned at birth, respectively. Baseline pubertal status was assessed using the parent-report Pubertal Development Scale (PDS), which evaluates physical markers of pubertal maturation such as growth of body hair, skin changes, and development of secondary sexual characteristics for males and females, respectively (Petersen et al., 1988). An overall summary score ranging from 1 to 5 is calculated to represent each participant’s pubertal stage, approximating the Tanner stages of pubertal development ((Tanner and Whitehouse, 1976), with higher scores indicating more advanced pubertal development. Parent-report scores were used in this study based on recommendations for use of the PDS in baseline ABCD data (Cheng et al., 2021).

## 5 Statistical analysis

### 5.1 Primary analysis

A logistic generalized linear mixed effects model (GLMM) with a binomial distribution and logit link was used to predict alcohol use status (non-initiators vs. initiators) from baseline BrainAGE, controlling for baseline age, sex, and pubertal status (fixed effects), and random intercepts accounting for family nested within data collection site. Numeric predictors (BrainAGE, age, pubertal status) were z-standardized (mean = 0, SD = 1) for interpretability and effect size comparability. Models were fitted via maximum likelihood with Laplace approximation using the *lme4* (v 1.1.37; Bates et al., 2015) package in R (*bobyqa* optimizer). Effects are reported as odds ratios (ORs) with 95% confidence intervals. Significance was assessed with two-sided tests at α = .05.

### 5.2 Secondary analysis

The primary model was repeated using the same parameters to predict alcohol behaviour subgroup classification (experimentation vs. binge drinking) from baseline BrainAGE.

### 5.3 Sensitivity analyses

Two sensitivity analyses were performed to assess the robustness of the primary model (non-initiators vs. initiators).

### 5.4 Sociodemographics and prior alcohol exposure

To assess whether observed differences in BrainAGE were independent of sociodemographic differences and/or prior alcohol exposure, the primary analysis was repeated with adjustment for covariates associated with brain structure and/or alcohol use in the ABCD sample. This included key sociodemographic variables (race, ethnicity, highest parental education, religious attitudes prohibiting alcohol use), as well as additional variables related to prenatal exposure and adolescent alcohol use (maternal alcohol use during pregnancy, participant reported sipping at baseline), as performed in previous work (Byrne et al., 2026). These variables were not interpreted as biological traits but included to reduce confounding related to structural and sociocultural factors known to affect neurodevelopmental trajectories and substance use risk. Further information on the variables used are reported in Table S4*.*

To determine the extent to which these additional variables accounted for the observed relationship between BrainAGE and alcohol initiation, overall and unique variance explained were examined in the fully adjusted model. Marginal and conditional R² were calculated using the *r.squaredGLMM* function from the MuMIn package (v1.48.19; Bartoń, 2026), where marginal R² (R²_m_) reflects variance explained by fixed effects alone and conditional R² (R^2^_c_) additionally incorporates variance attributable to the random effect of site. The unique contribution of each predictor was estimated by sequentially refitting the fully adjusted model with each predictor removed in turn and computing the difference in marginal R² relative to the full model, providing semi-partial R² estimates (ΔR²_m_) for each predictor.

### 5.5 Exclusion of other substance use from non-initiator group

Inclusion of participants in the non-initiating group who initiate other substances during follow-up may confound results, as initiation of these substances may reflect shared high-risk neural substrates with alcohol (Miller et al., 2024). Therefore, to assess whether BrainAGE differs at baseline between ‘true’ non-initiators and those who later initiated other substances, the GLMM was repeated excluding non-initiators who initiated cigarette, e-cigarette, or cannabis smoking at baseline and/or during follow-up, as reported during the SUI (see Table S4 for further detail on these measures).

## 6 Results

### 6.1 Participant characteristics

At baseline, participants who initiated alcohol use at or by wave 6 (n = 1176) differed on nearly all characteristics compared to non-initiators (n = 3639). Specifically, initiators were older than non-initiators, and a higher proportion were female, had been exposed to alcohol *in utero*, and reported alcohol sipping at baseline. In the non-initiating group, a greater proportion reported religious attitudes against alcohol compared to alcohol initiators, and groups differed in distribution of pubertal status, race, ethnicity, and highest caregiver education. Participant flowchart and characteristics are reported Fig. 1 and Table 1, respectively. To evaluate potential attrition bias, baseline characteristics were also compared between included non-initiators and participants excluded due to missing follow-up data (Table S2). Although some sociodemographic differences were present, baseline BrainAGE did not significantly differ between groups, suggesting that attrition was unlikely to bias these estimates.

**Table 1:** Participant characteristics of full study sample.

**Characteristic**	**Non-initiators (n = 3639)**	**Initiators (n = 1176)**	***p*****value**
BrainAGE^1,3^	0.52 (−0.56–1.70)	0.30 (−0.76–1.52)	< 0.001**
Age at baseline (years)^1,3^	9.94 (9.41–10.48)	10.35 (9.81–10.75)	< 0.001**
Sex^2,4^			< 0.001**
Female	1768 (49%)	638 (54%)	
Pubertal status at baseline^2,4^			< 0.001**
1	2040 (56%)	565 (48%)	
2	771 (21%)	271 (23%)	
3	779 (21%)	318 (27%)	
4	48 (1.3%)	21 (1.8%)	
5	< 10	< 10	
Race^2,5^			< 0.001**
American Indian/Alaska Native	18 (0.5%)	< 10	
Asian	87 (2.4%)	11 (0.9%)	
Black (African American)	454 (13%)	55 (4.8%)	
More than one race	380 (10%)	118 (10%)	
Native Hawaiian/Other Pacific Islander	< 10	< 10	
White	2610 (73%)	944 (83%)	
Missing/Other	87	38	
Ethnicity (Hispanic)^2,4^	663 (18%)	215 (18%)	0.9
Missing	46	10	
Caregiver education (highest)^2,5^			< 0.001**
Up to high school (no diploma)	131 (3.6%)	25 (2.1%)	
High school diploma/GED	236 (6.5%)	70 (6.0%)	
Some college	896 (25%)	260 (22%)	
Bachelor’s degree	1045 (29%)	288 (24%)	
Graduate school or professional degree	1326 (36%)	533 (45%)	
Missing	< 10	0	
Religious attitudes against alcohol^2,4^	2127 (59%)	517 (44%)	< 0.001**
Missing	46	14	
Prenatal alcohol exposure^2,4^	840 (24%)	427 (39%)	< 0.001**
Missing	203	61	
Alcohol sipping at baseline^2,4^	741 (20%)	517 (44%)	< 0.001**
Missing	0	14	

^1^ Median (IQR); ^2^ n (%); ^3^ Wilcoxon rank sum test; ^4^ Pearson’s Chi-squared test; ^5^ Fisher’s exact test. Counts less than 10 have been suppressed to protect participant confidentiality. * < 0.05; ** < 0.001.

To assess whether baseline age differences could bias follow-up comparisons, where alcohol use might be skewed in the initiator group because of older age at baseline, age distributions for non-initiators at wave 6 and age at first reported drink in the initiator group were examined (Figure S1). No meaningful skew toward older ages was observed in the initiator group relative to non-initiators, suggesting that older age in the initiator group was unlikely to bias the overall findings.

In contrast to the full sample, when comparing drinking subgroup characteristics, Experimenters (n = 461) and Bingers (n = 438) only differed on a small number of measures (Table 2).

**Table 2:** Participant characteristics within experimenter and binge drinking subgroups.

**Characteristic**	**Experimenters (n = 461)**	**Bingers (n = 438)**	***p*****value**
BrainAGE^1,3^	0.32 (−0.78–1.55)	0.19 (−0.83–1.41)	0.5
Age at baseline (years)^1,3^	10.29 (9.74–10.73)	10.42 (9.94–10.80)	0.009*
Sex^2,4^			0.2
Female	247 (54%)	252 (58%)	
Pubertal status at baseline^2,4^			0.5
1	218 (47%)	203 (46%)	
2	102 (22%)	100 (23%)	
3	127 (28%)	127 (29%)	
4	14 (3.0%)	< 10	
5	0	< 10	
Race^2,5^			0.4
American Indian/Alaska Native	< 10	< 10	
Asian	< 10	< 10	
Black (African American)	23 (5.1%)	16 (3.8%)	
More than one race	50 (11%)	40 (9.5%)	
Native Hawaiian/Other Pacific Islander	< 10	< 10	
White	373 (82%)	283 (86%)	
Missing/Other	< 10	16	
Ethnicity (Hispanic)^2,4^	63 (14%)	87 (20%)	0.013*
Missing	< 10	< 10	
Caregiver education (highest)^2,5^			0.068
Up to high school (no diploma)	< 10	14 (3.2%)	
High school diploma/GED	23 (5.0%)	25 (5.7%)	
Some college	90 (20%)	109 (25%)	
Bachelor’s degree	108 (23%)	110 (25%)	
Graduate school or professional degree	231 (50%)	180 (41%)	
Missing	0	0	
Religious attitudes against alcohol^2,4^	237 (52%)	195 (45%)	0.039*
Missing	< 10	< 10	
Prenatal alcohol exposure^2,4^	169 (40%)	164 (40%)	0.9
Missing	38	27	
Alcohol sipping at baseline^2,4^	185 (40%)	204 (47%)	0.051
Missing	0	0	

^1^ Median (IQR); ^2^ n (%); ^3^ Wilcoxon rank sum test; ^4^ Pearson’s Chi-squared test; ^5^ Fisher’s exact test. Counts less than 10 have been suppressed to protect participant confidentiality. * < 0.05; ** < 0.001.

### 6.2 BrainAGE model performance

In the original BrainAGE model validation, the model performed with a corrected (bias adjusted) MAE of 1.98 (Drobinin et al., 2022), aligning with the 1–2-year range typically reported by other adolescent BrainAGE models (Franke et al., 2012). In the current study, the model achieved an MAE of 2.28 prior to correction for age bias, and an MAE of 1.43 years after linear bias correction. The Pearson correlation between chronological age and raw predicted age was *r* = 0.24 (95% CI: 0.22–0.26, *p* < .001), and a negative correlation between raw BrainAGE and chronological age was observed (*r* = −0.39, 95% CI: −0.40 to −0.37, *p* < .001), indicating a systematic age bias that was addressed through bias correction prior to primary analyses. These findings correspond closely to the study by Whitmore et al. using the same model in ABCD data (2.32 uncorrected, 1.45 uncorrected), with minor differences likely attributable to differences in harmonization, including exclusion of participants with missing pubertal data. Similar to the findings reported by Whitmore et al. (2023), participants from the ABCD study exhibited, on average, higher (positive) BrainAGE estimates relative to the original Drobinin et al. (2022) reference sample, reflecting a tendency toward chronological age overestimation within the current cohort (Table 2).

### 6.3 Baseline BrainAGE as a predictor of alcohol initiation

**Fig. 2:**
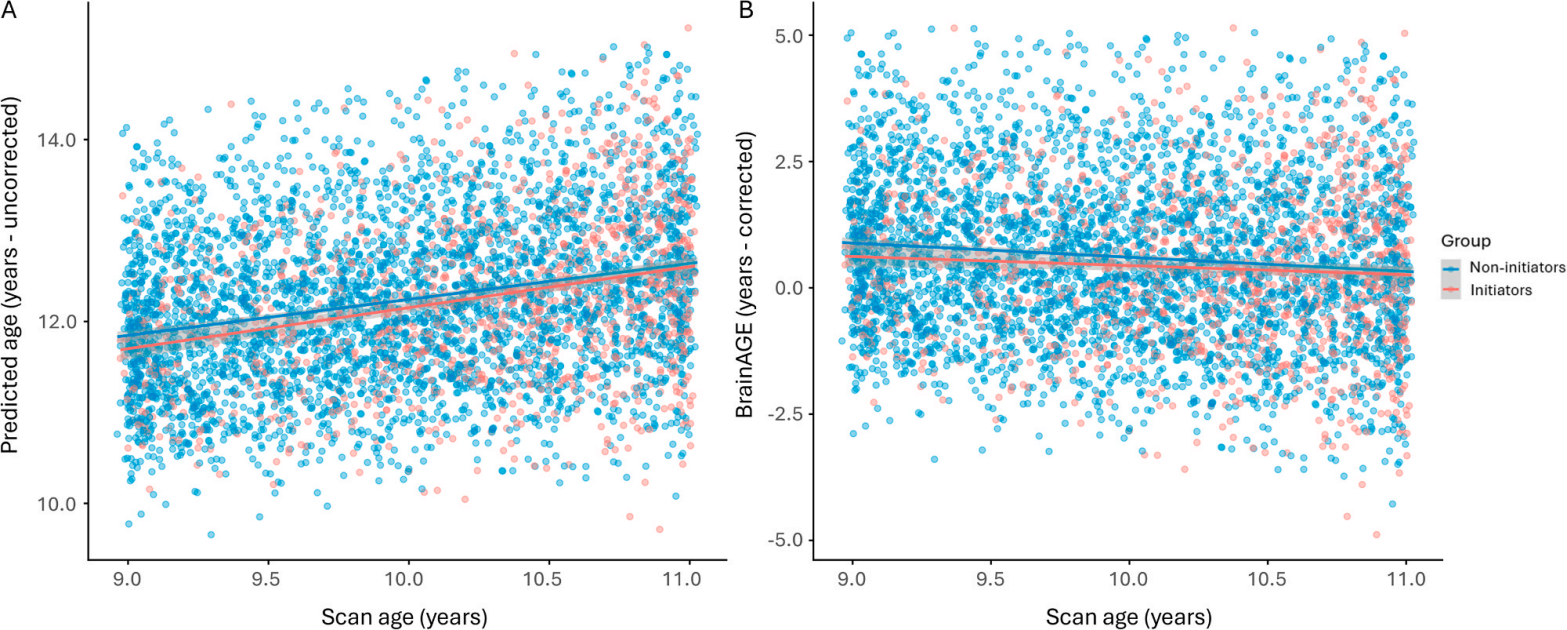
Scatterplots showing: (A) scan age compared to predicted age prior to age bias correction; (B) scan age compared to bias-corrected BrainAGE within the full study sample.].

Baseline BrainAGE (Fig. 2) significantly predicted alcohol use status by/at wave 6 (Table 3). Specifically, a one-standard-deviation increase (equivalent to 1.64 years) in BrainAGE was associated with an 8.7% decrease in the odds of alcohol use, holding other covariates constant. This equates to a 9.5% increase in the odds of alcohol use with a one-standard-deviation decrease in BrainAGE.

**Table 3:** Results from logistic GLMM predicting alcohol use status (non-initiation vs. initiation) from baseline BrainAGE.

**Predictor**	**OR**	**95% CI**	***p*****value**
BrainAGE (std)	0.913	0.844–0.987	0.023*
Age (std)	1.7	1.55–1.86	< .001**
Sex (female)	1.28	1.06–1.54	0.011*
Pubertal status (std)	1.04	0.95–1.14	0.377

OR = odds ratio; 95% CI = confidence intervals (odds ratio); std = standardized values. * < 0.05; ** < 0.001.

### 6.4 Baseline BrainAGE as a predictor of alcohol use behaviours

**Fig. 3:**
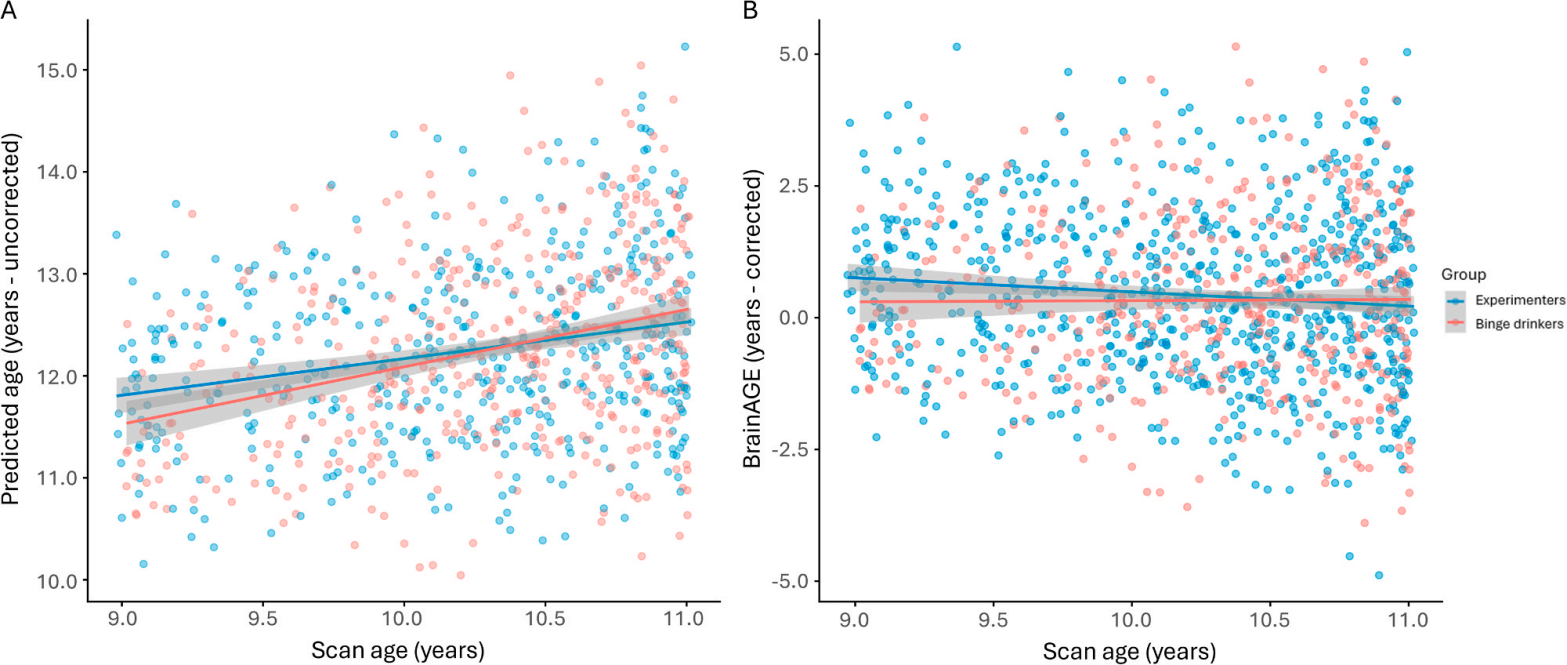
Scatterplots showing: (A) scan age compared to predicted age prior to age bias correction; (B) scan age compared to bias-corrected BrainAGE between the experimenter and binge drinking groups.].

Unlike the primary model, BrainAGE was not identified as a significant predictor of drinking behaviour (experimentation versus binge drinking; Fig. 3; Table 4).

**Table 4:** Results from logistic GLMM predicting alcohol use behaviour (experimentation vs. binge drinking) from baseline BrainAGE.

**Predictor**	**OR**	**95% CI**	***p*****value**
BrainAGE (std)	0.999	0.862–1.158	0.989
Age (std)	1.235	1.048–1.455	0.012*
Sex (female)	1.498	1.042–2.155	0.029*
Pubertal status (std)	0.82	0.671–1.003	0.053

OR = odds ratio; 95% CI = confidence intervals (odds ratio); std = standardized values. * < 0.05.

### 6.5 Sensitivity analyses

#### 6.5.1 Adjustment for secondary covariates

Following inclusion of secondary covariates in the primary model, no significant association between baseline BrainAGE and alcohol initiation status was identified (Table S3). Fixed effects in the omnibus model accounted for 18.8% of variance in alcohol initiation (R²_m_ = 0.19), increasing to 21% when random effects (site) were included (R²_c_ = 0.21). The negligible difference between marginal and conditional R² values indicate that site contributed relatively little additional explanatory variance beyond the fixed effects. Chronological age (ΔR²_m_ = 0.052) and prior alcohol sipping (ΔR²_m_ = 0.049) made the largest unique contributions, whereas BrainAGE contributed negligible unique explanatory variance (ΔR²_m_ = 0.0002). The attenuation of the BrainAGE effect in the fully adjusted model reflected both a shift in the point estimate toward the null (OR: 0.913–0.968) and a modest widening of confidence intervals. This pattern suggests these additional covariates partly account for the association between BrainAGE and alcohol use, rather than the null finding being solely attributable to reduced statistical power from exclusion of siblings (see Supplementary Materials) and participants with missing covariate data.

### 6.6 Exclusion of participants of initiating other substances

Following exclusion of participants in the non-initiating group who initiated other substances at baseline and reported use at any time during follow-up, BrainAGE was still identified as a significant predictor of alcohol use (Table S5).

## 7 Discussion

The present study investigated whether BrainAGE in late childhood could predict subsequent alcohol initiation and use behaviour in adolescence. Overall, ABCD participants showed positive BrainAGE estimates on average, reflecting a tendency toward chronological age overestimation relative to the model’s normative reference sample. However, lower BrainAGE (a one-standard-deviation decrease) relative to the non-initiating group was associated with a 9.5% increased likelihood of alcohol initiation at follow-up, independent of chronological age, sex, and pubertal status. However, these effects were not robust to adjustment for sociodemographic characteristics and prior alcohol exposure. Additionally, within the initiation group, BrainAGE did not differentiate between experimentation and more harmful binge drinking behaviours. Overall, these findings suggest lower BrainAGE may predate alcohol initiation in adolescence, however, this relationship likely reflects complex, context-dependent risk rather than a direct effect of brain structure alone, warranting further investigation into how these broader factors may shape brain structure and subsequent alcohol-use risk.

Results from our primary analysis suggest that lower BrainAGE (“less mature appearing” brains) in late childhood, relative to non-initiators, predicts subsequent alcohol initiation in adolescence. This contrasts with prior work identifying markers of accelerated neuromaturation, including prefrontal cortical thinning (Brumback et al., 2016, Squeglia et al., 2017) and regional volumetric increases (Miller et al., 2024), as predictors of alcohol use. This apparent discrepancy may partly reflect the limitations of region-of-interest approaches, which may not adequately capture the coordinated, network-level reorganization and structural co-maturation that characterizes brain development across childhood and adolescence (Liang et al., 2024). BrainAGE, by contrast, integrates information across distributed cortical and subcortical regions, capturing multivariate patterns of neuroanatomical variation that may not be evident in region-specific analyses. Accordingly, the lower BrainAGE observed among initiators may reflect an altered overall pattern of brain development, consistent with neurodevelopmental models proposing that disrupted maturation across interacting systems increases vulnerability to substance use (Casey and Jones, 2010, Steinberg, 2017). However, collapsing these features into a summary score from a single timepoint limits our ability to determine whether the observed BrainAGE difference reflects disruption across interacting systems, how individual regions or systems contribute to this difference, and whether these contributions evolve across development. Future work incorporating longitudinal and complementary network-level models alongside BrainAGE (such as structural covariance networks or connectome-based approaches) would help clarify whether disruption is coordinated or system-specific, more precisely mapping findings onto neurodevelopmental models of substance use vulnerability.

While these initial findings suggest that BrainAGE may predict alcohol initiation in adolescence, the absence of an association following covariate adjustment indicates that the observed relationship may largely reflect contextual risk rather than an independent biological propensity for alcohol use. Specifically, while BrainAGE explained negligible additional variance in the fully adjusted model, chronological age and prior alcohol sipping made the largest unique contributions to the prediction of subsequent initiation. This is consistent with the wider literature, where older age increases opportunities for alcohol use through greater autonomy and peer exposure, while prior sipping may reflect multiple familial, environmental, and individual risk factors – including socioeconomic disadvantage, adverse childhood experiences, and sensation seeking – that themselves are associated with subsequent alcohol use (Niklason et al., 2025, Nagata et al., 2023). This poses a significant challenge in the current context, as these broader influences have also been uniquely associated with variation in BrainAGE (Cohen et al., 2024, Rakesh et al., 2021, Dunlop et al., 2021). Consequently, our BrainAGE findings may partly reflect the neurodevelopmental correlates of contextual influences that also increase the likelihood of early alcohol exposure, rather than a distinct neuroanatomical pathway to initiation. More broadly, this underscores the need for caution when interpreting predictive neuroimaging markers of adolescent alcohol use, which may index shared developmental and environmental influences rather than specific biological vulnerability. Future mediation and causal inference analyses are therefore needed to clarify the independent and interrelated contributions of these factors to adolescent alcohol use (Visontay et al., 2024).

A further consideration of the current findings is the apparent lack of behavioural specificity. Although BrainAGE predicted alcohol initiation in the primary model, it did not differentiate between experimental and binge drinking behaviours, despite evidence that these may be neurobiologically distinct. For example, Rane et al. (2022) demonstrated that structural brain features in early adolescence (age 14) are more strongly predictive of later binge drinking than experimental use, suggesting the neural correlates underlying escalation may be dissociable from and more pronounced than those associated with initiation alone. It is possible that a composite measure such as BrainAGE, when indexed at ages 9–11, may lack the sensitivity to capture such nuances, particularly given the reduced statistical power from a smaller binge drinking subsample. Additional factors including family history of substance use, impulsivity, and psychopathology have been linked to both binge drinking independently of experimentation (Englund et al., 2008, Tschorn et al., 2021) and to BrainAGE in adolescence (Dunlop et al., 2021, Cropley et al., 2021), thus potentially obscuring group differences. Future work examining the relative contributions of such factors alongside BrainAGE would assist in determining its predictive utility across the spectrum of adolescent drinking behaviours, for which future releases of the ABCD study with larger alcohol use cohorts will be well-positioned to address.

### 7.1 Limitations

Strengths of this work include the use of a large, demographically diverse prospective sample, consideration of multiple sociodemographic and alcohol exposure variables, and stringent alcohol use criteria. However, BrainAGE estimation in childhood and adolescence remains a significant challenge (Whitmore and Beck, 2025). As BrainAGE collapses hundreds of regionally distinct and asynchronously maturing features into a single global deviation score, such measures may obscure tissue- and network-specific patterns associated with genetic, environmental, and/or psychosocial influences. Moreover, developmental BrainAGE estimates may be influenced by individual differences in pubertal maturation and sex-specific neurodevelopmental trajectories, which were not explicitly modelled within the BrainAGE framework used here. This is particularly relevant as research suggests pubertal development may show stronger associations with neurodevelopmental variation than chronological age alone (Whitmore and Beck, 2025, Holm et al., 2023b). Such limitations are exacerbated within cross-sectional frameworks, where normative BrainAGE trajectories in the paediatric population will be essential for informing interpretation of such metrics. While some longitudinal BrainAGE models are currently available (e.g. Beck et al., 2026, Whitmore et al., 2023), the development of an externally validated, comprehensive model integrating multimodal imaging features, sex and gender differences, and pubertal development will be pertinent to realizing this goal (Michael et al., 2026).

There are also some limitations to consider regarding the application of the Drobinin et al. (2022) model in the present context. While the model was trained across multiple independent developmental cohorts and has been externally evaluated in both independent and ABCD samples, its implementation here differed from the original model in some respects. First, 14 features from the original model were unavailable in the ABCD dataset and thus omitted from model implementation. Although these primarily comprised global summary metrics and small peripheral structure volumes rather than core cortical or subcortical morphometric measures, some influence on BrainAGE estimation cannot be excluded. Relatedly, bias correction parameters (slope and intercept) were derived from the external validation sample described by Drobinin et al. (2022), rather than estimated within ABCD, consistent with the implementation approach adopted by Whitmore et al. (2023) and avoiding potential circularity associated with recalibration in the target dataset. However, because the validation sample spanned a broader developmental age range (9–19 years) than the ABCD baseline cohort (9–11 years), and bias correction parameters may be sensitive to sample age distributions and feature composition, these parameters may not transfer perfectly to the current implementation. Together, the reduced feature set, broader developmental training range, and potential imperfect transfer of bias correction parameters may have contributed to the tendency toward chronological age overestimation observed in the current sample. Accordingly, BrainAGE estimates in the present study are best interpreted as relative deviations from the current reference sample rather than absolute indicators of biological brain maturation. Replication using alternative BrainAGE models would help determine the robustness of the observed association to model specification and implementation.

## 8 Conclusion

The current findings suggest that BrainAGE in late childhood may reflect potential risk for subsequent alcohol use initiation, but not use behaviours, in adolescence. However, this association likely reflects complex, context-dependent risk rather than a direct effect of brain structure alone. Further research tracking longitudinal trajectories of BrainAGE within a bidirectional framework to examine relationships between other genetic, environmental, and psychosocial variables will be essential for determining the role of BrainAGE in adolescent alcohol use.

## CRediT authorship contribution statement

**Rachel Visontay:** Conceptualization, Investigation, Methodology, Project administration, Writing – review & editing. **Emma Devine:** Conceptualization, Investigation, Methodology, Project administration, Writing – review & editing. **Hollie Byrne:** Conceptualization, Data curation, Formal analysis, Investigation, Methodology, Visualization, Writing – original draft, Writing – review & editing. **Louise Mewton:** Conceptualization, Funding acquisition, Methodology, Supervision, Writing – review & editing. **Andrew J Moore:** Methodology, Writing – review & editing. **Lindsay M Squeglia:** Conceptualization, Funding acquisition, Methodology, Supervision, Writing – review & editing. **Natasha E Wade:** Methodology, Writing – review & editing. **Joanna Jacobus:** Methodology, Writing – review & editing.

## Data statement

Data used in the preparation of this article were obtained from the Adolescent Brain Cognitive Development™ (ABCD) Study, held in the NIH Brain Development Cohorts Data Sharing Platform. This is a multisite, longitudinal study designed to recruit more than 10,000 children age 9–10 and follow them over 10 years into early adulthood. The ABCD Study® is supported by the 10.13039/100000002National Institutes of Health and additional federal partners under award numbers U01DA041048, U01DA050989, U01DA051016, U01DA041022, U01DA051018, U01DA051037, U01DA050987, U01DA041174, U01DA041106, U01DA041117, U01DA041028, U01DA041134, U01DA050988, U01DA051039, U01DA041156, U01DA041025, U01DA041120, U01DA051038, U01DA041148, U01DA041093, U01DA041089, U24DA041123, U24DA041147. A full list of supporters is available at https://abcdstudy.org/federal-partners.html. A listing of participating sites and a complete listing of the study investigators can be found at https://abcdstudy.org/consortium_members/. ABCD consortium investigators designed and implemented the study and/or provided data but did not necessarily participate in the analysis or writing of this report. This manuscript reflects the views of the authors and may not reflect the opinions or views of the NIH or ABCD consortium investigators.

## Funding

This work was supported by funding from the 10.13039/100000002NIH (R01AA030575, Mewton/Squeglia; and K24AA031052, Squeglia) and a National Health and Medical Research Council Centre of Research Excellence in the Prevention of Mental Illness and Substance Use (PREMISE Next Generation; APP2035308, Byrne). The authors have no financial disclosures or conflicts of interest to declare.

## Declaration of Competing Interest

The authors declare that they have no known competing financial interests or personal relationships that could have appeared to influence the work reported in this paper.

## Data Availability

The authors do not have permission to share data.
